# HIV-1 Coreceptor Usage Assessment by Ultra-Deep Pyrosequencing and Response to Maraviroc

**DOI:** 10.1371/journal.pone.0127816

**Published:** 2015-06-11

**Authors:** Christophe Rodriguez, Cathia Soulié, Anne-Geneviève Marcelin, Vincent Calvez, Diane Descamps, Charlotte Charpentier, Philippe Flandre, Patricia Recordon-Pinson, Pantxika Bellecave, Jean-Michel Pawlotsky, Bernard Masquelier, the ANRS AC11 Study Group

**Affiliations:** 1 National Reference Center for Viral Hepatitis B, C and delta, Department of Virology; Henri Mondor Hospital, University of Paris-Est, Créteil, France; 2 INSERM U955, Créteil, France; 3 Department of Virology, Pitié-Salpêtrière Hospital, Paris, France; 4 Department of Virology, Bichat-Claude Bernard Hospital, HUPNVS, Paris, France; 5 INSERM U943 UPMC, UMR-S 943, Paris, France; 6 Department of Virology, University Hospital of Bordeaux and UMR5234, University of Bordeaux, Bordeaux, France; Centro de Biología Molecular Severo Ochoa (CSIC-UAM), SPAIN

## Abstract

**Background:**

Maraviroc is an HIV entry inhibitor that alters the conformation of CCR5 and is poorly efficient in patients infected by viruses that use CXCR4 as an entry coreceptor. The goal of this study was to assess the capacity of ultra-deep pyrosequencing (UDPS) and different data analysis approaches to characterize HIV tropism at baseline and predict the therapeutic outcome on maraviroc treatment.

**Methods:**

113 patients with detectable HIV-1 RNA on HAART were treated with maraviroc. The virological response was assessed at months 1, 3 and 6. The sequence of the HIV V3 loop was determined at baseline and prediction of maraviroc response by different software and interpretation algorithms was analyzed.

**Results:**

UDPS followed by analysis with the Pyrotrop software or geno2pheno algorithm provided better prediction of the response to maraviroc than Sanger sequencing. We also found that the H34Y/S substitution in the V3 loop was the strongest individual predictor of maraviroc response, stronger than substitutions at positions 11 or 25 classically used in interpretation algorithms.

**Conclusions:**

UDPS is a powerful tool that can be used with confidence to predict maraviroc response in HIV-1-infected patients. Improvement of the predictive value of interpretation algorithms is possible and our results suggest that adding the H34S/Y substitution would substantially improve the performance of the 11/25/charge rule.

## Introduction

Human immunodeficiency virus (HIV) entry starts with the attachment of the viral envelope glycoprotein gp120 to the CD4-positive T-cell receptor and to either of two chemokine coreceptors: CCR5 or CXCR4 [1]. Maraviroc is an HIV entry inhibitor that prevents infection of CD4-positive T-cells by altering CCR5 conformation [2]. This therapy is poorly effective on viruses that use CXCR4 as an entry coreceptor. Thus, characterization of HIV tropism is important prior to deciding to use maraviroc [3]. The assessment of HIV tropism is classically based on two approaches. The first one is based on phenotypic assays [4], but the need for recombinant vectors in a culture system makes this method challenging in the clinical setting [5]. The genotypic approach is based on sequence analysis of the HIV V3 loop, the region involved in the interaction with the coreceptor that determines viral tropism. However, population sequencing has shown limitations in this setting [6].

HIV has a quasispecies distribution, characterized by the coexistence of closely related but distinct viral populations, including major and minor viral populations, in any given infected individual. Thus, pre-existing minor CXCR4 viral populations can be selected by maraviroc, expand and become predominant, ultimately leading to treatment failure, in spite of the exclusive detection of CCR5 viruses at baseline with inadequately sensitive methods. Previous studies have established that the presence of more than 2% of CXCR4 viral variants at baseline was predictive of maraviroc failure [7]. However, such sensitivity cannot be achieved by methods based on population sequencing. Cloning and sequencing would be sensitive enough only if a very large number of clones were generated, but this is not feasible in clinical practice. Thus, more sensitive genotyping techniques are needed to assess HIV tropism prior to initiating maraviroc therapy [8].

Next-generation sequencing methods, such as ultra-deep pyrosequencing (UDPS), have been developed to increase sequencing capacity while generating clonal sequences. They have been shown to be as sensitive as phenotypic methods [9,10]. An important challenge with this technology is the very large number of sequences generated, that requires complex dataset analyses in order that the information becomes clinically meaningful. Bioinformatics algorithms that differentiate CCR5 from CXCR4 viral variants classically use rules based on the presence of substitutions at positions 11 and 25 and the global charge of the V3 loop [11] or comparisons with phenotypic test databases. Statistical learning methods have been used to establish these rules, such as the geno2pheno[coreceptor] or geno2pheno[454] algorithms, for population sequencing and next-generation sequencing, respectively [12][13].

In this work, we used UDPS and different analytical approaches using statistical learning to assess HIV tropism and the capacity of baseline genotypic assessment to predict the therapeutic outcome on maraviroc treatment.

## Patients and Methods

### Patients

One hundred and thirteen patients with detectable HIV-1 subtype B RNA receiving highly active antiretroviral therapy (HAART) were enrolled in this study and treated with maraviroc in combination with optimized background therapy [14]. The characteristics of the patients are shown in Table 1. The study and informed consent were approved by the “Comité Consultatif de Traitement de l'Information dans la Recherche Scientifique et Médicale”and the “Commission Nationale Informatique et Libertés“. The patients had signed the Maraviroc Expanded Access Program (January 2007-August 2009) informed consent form and were specifically informed about their participation in the study.

**Table 1:** Characteristic of the study population.

Characteristics	Baseline (D0) (n = 111)	Maraviroc treatment M1 (n = 85)	Maraviroc treatment M3 (n = 79)	Maraviroc treatment M6 (n = 73)
**General**				
Male [%]	76.6			
Median age [yr (IQR)]	45.7 (42.1–51.2)			
Median CD4 cell [count/μL (IQR)]	257 (123–394)	NA	NA	338 (148–574)
Median plasma HIV-1 RNA level [log_10_ cp/mL]	4.2 (3.4–4.9)	2.0 (1.6–2.8)	1.8 (1.0–2.5)	1.8 (1.0–2.4)
HIV-1 subtype B [%]	100			
**Prior antiretroviral treatments**				
Median number of NRTIs (IQR)	6 (5–7)			
Median number of NNRTIs (IQR)	1 (1–2)			
Median number of PIs (IQR)	4 (3–6)			
Enfuvirtide [%]	45.0			
Raltegravir [%]	22.2			
**Coprescribed antiretroviral drugs**				
Raltegravir [%]	67.9			
Darunavir [%]	53.6			
Etravirine [%]	28.6			
Enfuvirtide [%]	17.0			

IQR, interquartile range; NA: non available

Patient’s sera were collected at baseline (D0) and month 1, 3 and 6 (M1, M3, M6) of maraviroc therapy. The patients were considered as responders to maraviroc if HIV RNA level (assessed by means of COBAS AmpliPrep/COBAS TaqMan HIV-1 Test, Roche Molecular Systems, Pleasanton, California) had decreased by at least 1.0 log relative to D0.

### HIV V3 loop sequence analysis

The sequence of the HIV V3 loop was determined at D0 by means of UDPS in the 113 patients, as previously described [7]. Briefly, three independent one-step nested PCR amplifications were performed using tagged primers. Amplicons were quantified, fixed onto microbeads, subjected to emulsion PCR and the beads were loaded onto picotiter plates for forward and reverse pyrosequencing by means of the GS-FLX Titanium Kit in the Genome Sequencer-FLX (454 Life Sciences, Roche Diagnostics Corp., Brandford, Connecticut).

### UDPS sequence analyses

First, the quality of the experiments was analyzed and demultiplexing was performed with our in-house software Pyroclass [15]. Four methods analyzing and reporting UDPS data were then compared. *Method 1*: consensus sequences were generated from UDPS data at D0 and used as a surrogate of population sequencing; they were interpreted by means of the geno2pheno[coreceptor] algorithm (Max-Planck-Institute), based on recommendations from the European Consensus Group on Clinical Management of HIV-1 Tropism Testing (false-positive rate: 5%) [16]. *Method 2*: the consensus sequences were interpreted by means of the 11/25/charge rule using a module included into PyroTrop, an in-house software developed for this application. *Method 3*: the full set of UDPS sequences was interpreted with geno2pheno[454], an adaptation of the geno2pheno algorithm to multiple sequence files, such as those obtained with UDPS [7]. This algorithm uses a cutoff value of 2% for the risk of maraviroc failure [17]. *Method 4*: the full set of UDPS sequences was interpreted with our in-house software PyroTrop. Briefly, the previously described PyroMute software [15] was modified to assess each validated V3 sequence by means of the 11/25/charge algorithm. PyroTrop uses three quality filters to eliminate unreliable sequences, including sequences with a PHRED quality score <20 [18], sequences not spanning the full-length HIV V3 loop, and sequences that are too divergent from the HXB2 strain reference HIV sequence (GenBank accession number K03455). The latter filter was used to eliminate eventual contaminations by non-HIV sequences. Then, PyroTrop ascribes each valid V3 sequence to either CCR5 or CXCR4 by means of the 11/25/charge algorithm [19] and provides the respective percentages of these two populations within the patient’s quasispecies (2% of CXCR4 variants was set as the detection threshold) [20].

The results are provided as the respective proportions of CCR5 and CXCR4 populations in the patient’s viral quasispecies, with a predefined lower limit of detection of 2% [7]. Additional modules were included in PyroTrop to assess the frequency of each amino acid at each position, allowing for “machine learning” on the full data set.

### Statistical analysis

Sensitivity, specificity, positive predictive value (PPV, defined as the capacity of the test to correctly predict a significant viral level decrease), negative predictive value (NPV, defined as the capacity of the test to predict the lack of significant viral level decrease) and Receiver Operating Characteristic (ROC) curves were compared for the 4 methods by means of McNemar test and area under the curves (AUROC) comparisons. In addition, supervised classification using random forest, regression using lasso, and decision tree methods were used to assess the relationship between amino acid polymorphisms and the response to maraviroc. For each selected candidate polymorphism, ROC curves were plotted and the AUROCs were compared by means of bootstrap methods using an AUROC of 0.5 as non-predictive [21]. Analyses were made with packages randomForest, rpart, MASS, ROCR, cluster, pvclust, glmnet and pROC in R language software by means of RGui (64-bit) (v2.14.1) [22,23].

## Results

HIV RNA could not be amplified in 11 patients at D0. Ten of them had an HIV RNA level below 1000 copies/ml; the remaining one had an HIV RNA level of 4.6 log copies/ml but could not be amplified on three repeat tests. Pyrosequencing data of good quality were obtained in the remaining 102 patients at D0; 85, 79 and 73 of them had samples available for HIV RNA level determination at M1, M3 and M6, respectively. Overall, 16%, 13% and 12% of patients were considered as non-responders (<1 log HIV RNA level decrease) at M1, M3 and M6, respectively. The HIV RNA kinetics in responders is shown in S1 Fig.

UDPS generated a total of 626,874 sequences (mean coverage 6146±298 per sample), among which 539,096 were valid for geno2pheno[454] analysis (mean coverage 5285±290 per sample; rejection rate: 14.3%) and 461,044 for PyroTrop analysis (mean coverage 4520±306 per sample; rejection rate: 26,6%). Most of the removed sequences were rejected because the coverage of the V3 loop was partial (criterion used by all methods). In addition, PyroTrop rejected poor quality sequences or sequences which acceptable quality on only a portion of the V3 loop.

Concordance between the four methods is shown in Table 2 for the 102 patients with available UDPS sequences at D0. Best concordance (94.1%) was obtained with the two methods (consensus and UDPS) using Pyrotrop software.

**Table 2:** Concordance between the 4 methods used to analyze HIV tropism in the 102 patients with UDPS sequences of good quality at D0.

	Consensus/g2p % (n)	Consensus/Pyrotrop % (n)	UDPS/g2p % (n)	UDPS/Pyrotrop % (n)
Consensus/g2p		76.5% (78)	75.5% (77)	71.6% (73)
Consensus/Pyrotrop			87.3% (89)	94.1% (96)
UDPS/g2p				91.2% (93)
UDPS/Pyrotrop				

Consensus means that the consensus of UDPS sequences has been used as a surrogate of population sequencing; UDPS means that the full set of pyrosequencing sequences has been used; g2p means that the geno2pheno algorithm has been used for analysis; Pyrotrop means that our in-house software has been used for analysis.

The performance of the four methods in predicting the virological response to maraviroc was compared. As shown in Table 3, sensitivity was slightly higher for methods based on analysis of the full set of UDPS sequences, whereas the best specificity was achieved with UDPS data analyzed by means of Pyrotrop. The best positive predictive value was provided by Pyrotrop analysis, whereas the best negative predictive value was achieved with the full set of UDPS data (Table 3). In addition, Table 4 shows the results obtained with the 4 methods in samples with discrepant predictions of virological outcome.

**Table 3:** Sensitivity, specificity, positive and negative predictive values (PPV and NPV) of the 4 methods used to predict HIV RNA level decrease >1.0 log at M1, M3 and M6 on maraviroc therapy in the patients with available HIV RNA levels at D0 and M1.

	Sensitivity			Specificity			Positive predictive value			Negative predictive value		
**Virologic response**	**M1**	**M3**	**M6**	**M1**	**M3**	**M6**	**M1**	**M3**	**M6**	**M1**	**M3**	**M6**
**Consensus/g2p**	84.1%	86.0%	86.5%	9.1*%	4.5*%	4.8*%	72.6%*	70.0%*	69.2%*	16.7%*	11.1%*	12.5%*
**Consensus/Pyrotrop**	86.4%	89.3%	89.9%	25.0%	25.0%	25.0%	95.9%	95.7%	95.4%	8.3%*	11.1%*	12.5%*
**UDPS/g2p**	88.7%	89.7%	90.5%	28.6%	18.2%	20.0%	86.3%	87.1%	87.7%	33.3%	22.2%	25.0%
**UDPS/Pyrotrop**	88.0%	90.1%	90.9%	30.0%	25.0%	28.6%	90.4%	91.4%	92.3%	25.0%	22.2%	25.0%

*: p<0.05 versus all of the other techniques

The PPV was defined as the capacity of the test (CCR5 tropism) to correctly predict a significant viral level decrease; the NPV was defined as the capacity of the test (CXCR4 tropism) to predict the lack of significant viral level decrease. Consensus means that the consensus of UDPS sequences has been used as a surrogate of population sequencing; UDPS means that the full set of pyrosequencing sequences has been used; g2p means that the geno2pheno algorithm has been used for analysis; Pyrotrop means that our in-house software has been used for analysis.

**Table 4:** Results of the 4 technical/analytical methods in patients with discrepant results, and HIV-1 RNA level change at M1.

Sample	Consensus/g2p (FPR 5%)	Consensus/Pyrotrop	UDPS/g2p	UDPS/PyroTrop	HIV RNA level decrease at M1 (log cp/mL)
1	CCR5*	CCR5*	3.4%^§^	0%*	-2.6
2	CCR5*	CCR5*	3.3%^§^	3.1%^§^	-0.4
3	CXCR4^§^	CCR5*	0.2%*	0.03%*	-2.4
4	CCR5^§^	CCR5^§^	10.8%*	8.9%*	+0.2
5	CXCR4^§^	CCR5*	4.3%^§^	0.1%*	-2.4
6	CXCR4^§^	CCR5*	0.1%*	0.3%*	-2.7
7	CXCR4^§^	CCR5*	11.9%^§^	0.1%*	-2.5
8	CXCR4^§^	CCR5*	0.7%*	0%*	-1.7
9	CXCR4^§^	CCR5*	0.2%*	0.2%*	-2.3
10	CCR5^§^	CCR5^§^	0.1%^§^	0.02%^§^	-0.03
11	CXCR4^§^	CXCR4^§^	90.4%^§^	90.3%^§^	+0.8
12	CXCR4^§^	CXCR4^§^	0.7%*	90.6%^§^	-2.3
13	CXCR4^§^	CXCR4^§^	97.5%^§^	97.6%^§^	-2.3
14	CXCR4^§^	CCR5*	0.5%*	0.4%*	-2.9
15	CXCR4^§^	CCR5*	1.3%*	0%*	-2.6
16	CCR5^§^	CCR5^§^	0%^§^	0%^§^	-0.2
17	CCR5^§^	CCR5^§^	0%^§^	0.01%^§^	+0.3
18	CCR5^§^	CCR5^§^	0%^§^	0.02%^§^	-2.5
19	CXCR4^§^	CCR5*	20.3%^§^	33.3%^§^	-2.7
20	CXCR4^§^	CCR5*	0.1%*	0%*	-2.7
21	CCR5*	CCR5*	3,20.0%^§^	3.7%^§^	-2.4
22	CXCR4^§^	CCR5*	0%*	0%*	-2.4
23	CCR5*	CCR5*	0%*	9.7%^§^	-1.9
24	CCR5*	CCR5*	30.6%^§^	0.4%*	-2.1
25	CXCR4^§^	CCR5*	0.5%*	0.06%*	-2.4
26	CXCR4^§^	CCR5*	97.7%^§^	0.2%*	-1.9
27	CXCR4^§^	CCR5*	2.5%^§^	0%*	-0.5
28	CCR5^§^	CCR5^§^	0%^§^	0%^§^	-0.5
29	CXCR4^§^	CCR5*	0.2%*	0.08%*	-3.0
30	CXCR4^§^	CCR5*	98.7%^§^	0.9%*	-3.6
31	CXCR4^§^	CCR5*	1.4%*	0%*	-2.3
32	CCR5^§^	CCR5^§^	0%^§^	0.4%^§^	-0.7
33	CXCR4^§^	CCR5*	0.2%*	0%*	-1.9
34	CCR5^§^	CCR5^§^	0%^§^	0%^§^	+0.08

^§^ indicates that the method correctly predicted maraviroc response

* that it incorrectly predicted maraviroc response. FPR: false-positive rate.

**Fig 1:**
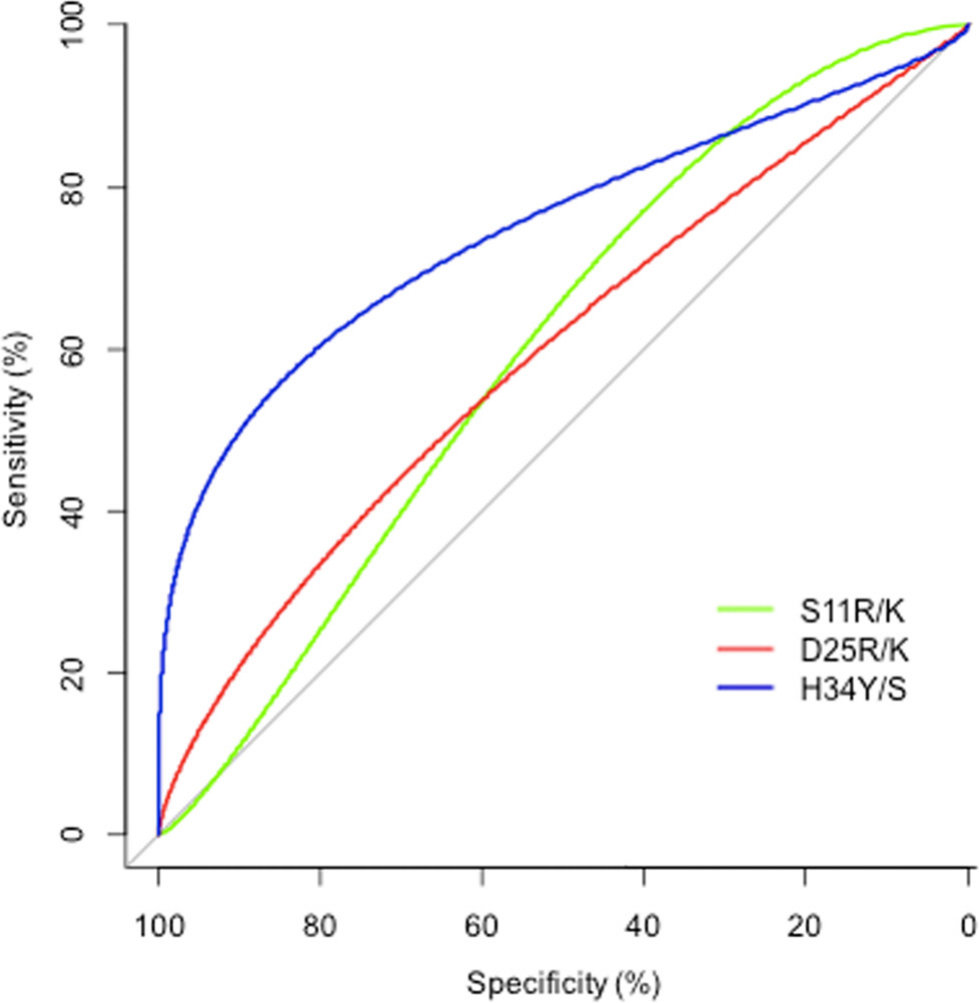
ROC curves testing the ability of individual amino acid substitutions in the HIV V3 loop to predict the response to maraviroc. p values were <0.011 for H34Y/S, = 0.13 for S11R/K and = 0.08 for D25R/K.

The ability of each observed amino acid at each position in the V3 loop to predict maraviroc response was assessed by means of machine learning. Position 34, particularly the H34Y/S substitution, was the only one found to be significantly associated with the response to maraviroc by means of a decision tree approach (bootstrap >95%, p<0.011) (Fig 1). Substitutions at positions 11 and 25, which are used in the 11/25/charge rule, also individually predicted the response, but significance was not reached (p = 0.13 and p = 0.08, respectively) (Fig 1). H34Y/S was present in 23% of the maraviroc responders, in whom it represented on average 79.6% of the viral quasispecies population. In contrast, it was not detectable at significant levels in any of the non-responders to maraviroc (p = 0.01).

## Discussion

In this study, we assessed the performance of methods based on consensus (mimicking population sequencing) and UDPS sequence analysis, followed by geno2pheno or Pyrotrop data analysis, to predict the response to maraviroc in HIV-1 subtype B-infected patients under HAART therapy. Our results show that using UDPS instead of population sequencing improved specificity and the positive and negative predictive values, while the use of our in-house software Pyrotrop for UDPS sequence analysis further improved specificity and the positive predictive value as compared to geno2pheno. Overall, the rates of correct prediction of maraviroc response were significantly improved when using UDPS and analyzing the data with Pyrotrop, versus consensus sequence and the geno2pheno algorithm.

It is currently possible to assess the distribution of HIV entry coreceptors and predict the response to maraviroc by means of sequencing methods, most often based on Sanger sequencing, now based on UDPS, using various software/algorithms. Recent studies have suggested that UDPS improved the prediction of maraviroc response when used instead of Sanger sequencing [8,24]. Nevertheless, it has also been shown that different algorithms/software may yield different results, emphasizing the need for standardization and the combined use of complementary approaches [10]. In this study, we confirmed the better performance of UDPS as compared to Sanger sequencing and validated our in-house software Pyrotrop as an alternative to geno2pheno to optimize analysis of UDPS data. Interestingly, the negative predictive value of UDPS sequencing was lower in our study than in a previous one comparing Sanger sequencing and UDPS [8]. This might be due to the lower proportion of nonresponders in our study. Also, other reasons than tropism such as poor observance, pharmacological factors, etc, may, at least in part, explain the lack of response to maraviroc.

In addition, our analysis suggests that predictions based on the 11/25 charge rule can be improved by including other parameters in the algorithm. Indeed, we found that the presence of an H34S/Y substitution was significantly associated with CCR5 genotropism. This association was even stronger than the individual associations of substitutions at positions 11 or 25 and was not fully reflected by charge changes. Thus, H34S/Y deserves to be included into a new algorithm. It must be emphasized, however, that all patients in the study were infected with HIV-1 subtype B and maraviroc failure occurred in only 16% of them at M1. Thus, this approach will require prospective confirmation in other, larger cohorts of patients infected with different HIV subtypes and followed for more than 6 months on treatment.

It is interesting to note that the H34S/Y substitution has been recently reported to play an important role in V3 affinity for both CCR5 and CXCR4, in particular when combined with a Q32K substitution [25]. In our study, only half of the patients with an H34Y/S substitution also had a Q32K substitution and no relationship with the response was found. The predictive role of this substitution, alone or combined with H34S/Y will need to be further explored in larger series of patients. We looked at the frequency of these substitutions in the Los Alamos Database of HIV sequences (www.hiv.lanl.gov/content/sequence/HIV/mainpage.html). As shown in Table 5, 60.4% of the HIV sequences available in the database were wild-type, 25.0% had one (17.3%) or two (7.7%) of these substitutions. When restricting the analysis to HIV-1 subtype B sequences, 69.2% had the wild-type sequence, 16.6% had one or two of these substitutions. These findings suggest that using this additional sequence parameter in algorithms for the determination of HIV tropism could benefit to a substantial number of patients. Nevertheless, the database is essentially based on population sequencing and does not provide information on the respective frequencies of the different viral populations in the quasispecies.

**Table 5:** Frequency of H34Y/S and Q32K substitutions in the Los Alamos database.

Genotype	Motif (amino acid positions 31–35)	% in all sequences (N = 172,550)	% in all subtype B sequences (N = 98,510)
Wt	RQAHC	60.4%	69.2%
H34Y	RQAYC	9.5%	6.8%
Q32K	RKAHC	7.6%	4.8%
Q32K+H34Y	RKAYC	7.6%	4.8%
H34S	RQASC	0.2%	0.1%
Q32K+H34S	RKASC	0.1%	0.1%
Others	-	14.6%	14.2%

In conclusion, UDPS is a powerful tool that can be used with confidence to predict maraviroc response in HIV-1-infected patients. Different data analysis tools can be used and we validated here the performance of our in-house Pyrotrop software that may represent a performing alternative to geno2pheno. Improvement of the predictive value of the algorithm used is possible and our results suggest that adding the H34S/Y substitution would substantially improve the performance of the 11/25 charge rule with these new approaches.

## Supporting Information

S1 Fig HIV RNA level kinetics on maraviroc therapy in responders. Only one patient experienced a viral rebound at M6.
(DOCX)
