# Supplementary material for: HIV-1 Coreceptor Usage Assessment by Ultra-Deep Pyrosequencing and Response to Maraviroc
Source: PLoS One. 2015 Jun 11;10(6):e0127816. doi: 10.1371/journal.pone.0127816 (PMC4466260; doi:10.1371/journal.pone.0127816)
Supplement: S1 Fig — Only one patient experienced a viral rebound at M6. (DOCX) [file pone.0127816.s001.docx]

Supplementary Figure : HIV RNA level kinetics on maraviroc therapy in responders. Only one patient experienced a viral rebound at M6.
